# Modeling thalamic dynamics with a network of integrate and fire neurons

**DOI:** 10.1186/1471-2202-16-S1-P76

**Published:** 2015-12-18

**Authors:** Alessandro Barardi, Alberto Mazzoni, Jordi Garcia-Ojalvo

**Affiliations:** 1 Department of Physics and Nuclear Engineering, Universitat Politecnica de Catalunya, Terrassa, 08222 Spain; 2 Department of Experimental and Health Sciences, Universitat Pompeu Fabra, Barcelona, Barcelona, 08003 Spain; 3 The BioRobotics Institute, Scuola Superiore Sant'Anna, Pontedera, 56026, Italy

## 

Spindle oscillations are rhythmic oscillations at 8-14 Hz originated in the thalamus during slow-wave sleep. The generation of this rhythm is due to the rebound bursting properties of thalamo-cortical (TC) relay cells, which are mutually connected with the thalamic reticular neurons (RE). Several experiments have shown that during sleep TC neurons can produce bursts of action potentials due to disinhibition from RE neurons. These bursts in turn trigger an inhibitory rebound in TC neurons thus giving rise to an endogenous oscillatory activity [1,2]. Various sophisticated HH-based models have described in detail the electrophysiological properties of these neurons and replicate most of the experimental observations made in-vivo and in-vitro [2]. However these types of models are computationally very expensive, thus raising the question of whether this dynamical behavior can be reproduced in simpler models.

To address this question, we used an adaptive exponential integrate and fire model (aEIF) [3] and studied the conditions under which spindle oscillations appear in large neuronal networks. The aEIF model can reproduce the rebound bursting properties of thalamic cells and has been reported to replicate the self-sustained oscillatory behavior in a minimal 4-neuron circuit [4]. In this minimal motif, RE cells exhibit periodic sequences of bursts at 10 Hz (spindles) and recruit TC neurons, which oscillate in anti-phase at half the frequency (cycle skipping) tightly correlated with IPSP bursts. However, it is unclear whether these spindle oscillations are preserved when the neurons are interconnected in a larger network, while keeping their intrinsic properties.

In order to examine this issue, we have systematically extended the size of the network of TC and RE neurons, and checked whether the features of the above-described oscillatory activity hold out. We found the reciprocal interaction between TC and RE to be essential for sustaining this activity, coherently with experimental results [5]. Furthermore, some of the RE neurons exhibit periodic bursting at the spindle frequency, provided they are not connected to TC neurons directly, but only indirectly through local inhibitory connections with RE neighbors. Interestingly, this behavior seems to require that the RE layer has a certain degree of clustering. Under these conditions, a few TC neurons in the network respond with a burst every two cycles (cycle skipping). The fact that most TC do not exhibit cycle skipping could associated with a large strength of GABA IPSPs impinging on them, according to results of HH kinetic models [6].

In summary, our aEIF network model provides a computationally efficient description of the dynamical features of the thalamus while preserving the properties of the individual neurons.
